# Targeting FRET-Based Reporters for cAMP and PKA Activity Using AKAP79

**DOI:** 10.3390/s18072164

**Published:** 2018-07-05

**Authors:** Nshunge Musheshe, Miguel J. Lobo, Martina Schmidt, Manuela Zaccolo

**Affiliations:** 1 Department of Molecular Pharmacology, University of Groningen, PO Box 72, 9700 AB Groningen, The Netherlands; nshunge.musheshe@dpag.ox.ac.uk (N.M.); m.schmidt@rug.nl (M.S.); 2 Department of Physiology, Anatomy and Genetics, University of Oxford, Oxford OX1 2JD, UK; miguel.lobo@dpag.ox.ac.uk; 3 Groningen Research Institute for Asthma and COPD, GRIAC, University Medical Center Groningen, University of Groningen, PO Box 72, 9700 AB Groningen, The Netherlands

**Keywords:** fluorescence resonance energy transfer (FRET), AKAP79, cAMP, protein kinase A (PKA), phosphatases, adrenergic signaling, real-time imaging

## Abstract

Fluorescence resonance energy transfer (FRET)-based sensors for 3′–5′cyclic adenosine monophosphate (cAMP) and protein kinase A (PKA) allow real-time imaging of cAMP levels and kinase activity in intact cells with high spatiotemporal resolution. The development of FRET-based sensors has made it possible to directly demonstrate that cAMP and PKA signals are compartmentalized. These sensors are currently widely used to dissect the organization and physiological function of local cAMP/PKA signaling events in a variety of cell systems. Fusion to targeting domains has been used to direct the sensors to a specific subcellular nanodomain and to monitor cAMP and PKA activity at specific subcellular sites. Here, we investigate the effects of using the A-kinase anchoring protein 79 (AKAP79) as a targeting domain for cAMP and PKA FRET-based reporters. As AKAP79 interacts with PKA itself, when used as a targeting domain, it can potentially impact on the amplitude and kinetics of the signals recorded locally. By using as the targeting domain wild type AKAP79 or a mutant that cannot interact with PKA, we establish that AKAP79 does not affect the amplitude and kinetics of cAMP changes or the level of PKA activity detected by the sensor.

## 1. Introduction

It is now recognized that signaling by the second messenger 3′–5′cyclic AMP (cAMP) is compartmentalized in subcellular nanodomains. The activation of different Gs protein coupled receptors leads to elevation of cAMP in distinct and confined subcellular sites. This results in the activation of limited subsets of protein kinase A (PKA) enzymes, which in turn phosphorylate one or more defined protein targets, resulting in the appropriate cellular response to the specific extracellular stimulus [1]. A-kinase anchoring proteins (AKAPs), a family of structurally unrelated scaffolding proteins, are localized to different subcellular sites and contribute to compartmentalization of signaling by binding PKA and targeting the enzyme in proximity of its specific targets [2]. Phosphodiesterases (PDEs)—the enzymes that hydrolyze cAMP to AMP—are a large superfamily of metallohydrolases that includes over 50 isoforms. PDEs can also be targeted to specific subcellular sites by either protein–lipid or protein–protein interactions. At their subcellular anchor sites, PDEs act as sinks for cAMP thus preventing the homogeneous distribution of the second messenger in the cell and contributing to signal compartmentalization [3]. The PKA signal is terminated by phosphatases, which dephosphorylate PKA targets [4], and in addition to determining the duration of PKA signaling, they can also contribute to compartmentalization by dampening the effects of any inappropriate PKA activation that may occur outside the relevant nanodomain.

The development of fluorescence resonance energy transfer (FRET)-based sensors has been instrumental in demonstrating compartmentalization of the cAMP/PKA signaling pathway [5,6,7,8,9]. These sensors are genetically encoded and can be expressed in living cells, allowing for real-time imaging of cAMP levels and PKA activity in the intact intracellular environment [10]. Fusion of a targeting domain to cAMP or PKA FRET-based sensors has been recently used to direct the sensor to a specific subcellular site for accurate monitoring of signaling events at that specific nanodomain. This has been successfully exploited to study compartmentalization of cAMP [6,7,11] and PKA activity [8,12]. For subcellular targeting, a number of investigations have relied on fusion of the FRET sensor to AKAPs [9,13,14] and most commonly AKAP79 [9,13,15,16]. As other AKAPs, AKAP79 contains an amphipathic α-helix, which binds the dimerization/docking (D/D) domain (amino acid 1–45) of the PKA RII subunits [17]. AKAP79 also has a plasma membrane localization signal that binds to phosphatidylinositol-4,5 biphosphate (PIP2) [18]. AKAP79 forms a complex with multiple signaling molecules. In addition to PKA, it interacts with protein kinase C (PKC) [19] and Ca^2+^-dependent protein phosphatase 2B (PP2B) [4]. In addition, in cardiac mycoytes, AKAP79 forms a complex with β-adrenergic receptors (β-AR), the adenylyl cyclases 5 and 6 (AC5/6), and L-type Ca^2+^ channels (LTCC) [20]. AKAP79-anchored PKA has been shown to phosphorylate AC5/6 to dynamically suppress cAMP synthesis [21,22]. In addition, AKAP79-anchored PKA has been shown to participate in a negative feedback loop, whereby local increase in cAMP activates the anchored PKA, which in turn phosphorylates and activates long isoforms of PDE4 [23,24]. Activated PDE4 more effectively degrades local cAMP leading to reduced PKA activation [15,25,26]. Although PDEs do not seem to necessarily interact with AKAPs directly, PDE4D5 has been suggested to be an essential component of the AKAP79-based complex [15,27,28]. Given the complexity of the signalosome organized by AKAP79, the use of this scaffolding protein for targeting cAMP or PKA FRET-based sensors to the plasma membrane may result in disruption of the nanodomain as the overexpressed targeted sensor may be expected to bring to the targeting site additional kinases and phosphatases that may alter local cAMP levels and PKA activity, resulting in artefactual signals. In this study, by using a wild-type AKAP79 and a mutant version that cannot bind PKA RII [17] we investigate the impact of using AKAP79 as a targeting domain on local cAMP/PKA signals in neonatal rat ventricular myocytes (NRVMs), a cell type that expresses endogenous AKAP79 and where AC5/6 and PDE4 are the most abundantly expressed adenylyl cyclase and phosphodiesterase isoforms, respectively. Our results show that PKA anchoring to AKAP79-targeted sensors does not significantly affect the level and kinetics of cAMP nor the PKA activity detected locally.

## 2. Materials and Methods

### 2.1. Site-Directed Mutagenesis

To generate the AKAP79_Mut_ construct, the PKA-binding site of the full-length AKAP79 protein was mutagenized by introducing leucine to proline substitutions in position Leu391 and Leu392 [17] using the Q5 Site-Directed Mutagenesis Kit (New England Biolabs, Hertz, UK)). The forward primer, 5′-GAAACACCCCCAATTGAAACAG-3′, and the reverse primer, 5′-ATATTGTTCTGAAGTTCTATCCTC-3′, were employed. The mutant AKAP79 was cloned in frame at the 5′ end of the CUTie [9] or AKAR4 [29] sensor encoded in the pcDNA3.1 vector (Addgene, Cambridge, MA, USA).

### 2.2. Isolation and Culture of Cardiomyocytes

Neonatal rat ventricular myocytes (NRVMs) from 1–3-day-old Sprague Dawley rats were cultured and isolated as described in [5]. In brief: left ventricles were dissociated enzymatically. The cell suspension was then plated on 24-mm glass coverslips coated with laminin (20 μg/mL). Cells were cultured in Dulbecco’s modified Eagle’s medium (DMEM) supplemented with 10% horse serum (HS) and 5% newborn calf serum (NCS). After 24 h in culture, the medium was replaced with DMEM supplemented with 5% HS and 0.5% NCS. The cells were then transiently transfected with TransFectinTM Lipid Reagent (BIO-RAD 170-3351) using 3 μg of DNA per well. The efficiency of transfection was about 20%. All experiments were performed at 37 °C, 36–48 h after transfection in 4-(2-hydroxyethyl)-1-piperazineethanesulfonic acid (HEPES)-buffered saline supplemented with 1 g/L of glucose.

### 2.3. Pull Down Experiments and Western Blotting

For NRVMs, 6 × 10^6^ cells were plated onto 2 × 10 cm dishes coated with laminin (20 μg/mL). For transfection, 15 μg plasmid DNA was mixed with TransFectin transfection reagent following manufacturer instructions. A total of 24 h after transfection, the cells were washed with 1 × ADS buffer (106 mM NaCl, 20 mM HEPES, 0.8 mM NaH2PO4, 5.3 mM KCl, 0.4 mM MgSO4, 5 mM glucose) and lysed for 5 min on ice in Ripa buffer (Sigma-Aldrich, Gillingham, UK) for the samples expressing the respective AKAP79-targeted constructs. The lysis buffers were supplemented with complete ethylenediaminetetraacetic acid (EDTA)-free protease inhibitor cocktail tablets (Roche Diagnostics Limited, West Sussex, UK) and phosphatase inhibitor tablets. The lysed cells were then collected and rotated for 20 min at 4 °C. The samples were then centrifuged for 10 min at 10,000 rpm at 4 °C to remove insoluble material. Total protein was quantified using a Micro BCA Protein Assay Kit (Pierce Biotechnology Inc., Rockford, IL, USA). 500 μg of total protein was rotated for 2 h at 4 °C with 25 μL of agarose beads coated with a monoclonal anti-GFP antibody (GFP-Trap_A, gta-10, ChromoTek GmbH, Planegg-Martinsried, Germany). The samples were then centrifuged at 2000 rpm for 1 min, and the supernatant discarded. The beads were washed at least four times with ice cold Ripa buffer. Bound proteins were eluted in 25 μL 2 × SDS loading buffer (Life Technologies, Carlsbad, CA, USA) and denatured at 95 °C for 5 min. Pulled down proteins were run on 4–12% Bolt Bis-Tris Plus Gels (Thermo Fisher Scientific, Waltham, MA, USA). The proteins were then transferred onto hybond-*p*, 0.45 μm polyvinylidene difluoride (PVDF) membrane (Amersham, GE Healthcare Life Sciences, Little Chalfont, UK). After the transfer, the membranes were blocked for 1 h at room temperature in 5% skim milk (Sigma Aldrich, UK). They were then incubated overnight at 4 °C with the following antibodies: Adenyl cyclase V/VI (C17) (Santa Cruz Biotechnology, Dallas, TX, USA, at 1:200), PKA RIIα (Santa Cruz Biotechnology, Dallas, TX, USA, at 1:1000). After at least five washes with Tris-buffered saline (TBS)-0.5% Tween20 (Alfa Aesar, Haverhill, MA, USA), the membranes were incubated at room temperature for 1 h with the respective horseradish peroxidase conjugated secondary antibodies (at 1:3000) and detected with enhanced chemiluminescence (ECL) western blotting detection kit (Thermo Fisher Scientific, Waltham, MA, USA). The blots were stripped with stripping buffer (Thermo Fisher Scientific, Waltham, MA, USA) and reprobed with anti-glyceraldehyde-3-phosphate dehydrogenase (GAPDH) antibody (sc-1666574, Santa Cruz Biotechnology, Dallas, TX, USA, used at 1:3000). In order to control for efficiency of the pull down, an anti-green fluorescent protein (GFP) antibody (sc-9996, Santa Cruz Biotechnology, Dallas, TX, USA, at 1:1000) was used.

### 2.4. FRET Imaging

FRET imaging experiments were performed 36–48 h after infection of the neonatal rat ventricular myocytes (NRVMs) with the pcDNA3.1 vector carrying each targeted sensor, as described in [30]. The cells were maintained at room temperature in a modified Ringer solution (140 mM NaCl, 3 mM KCL, 2 mM MgCL_2_ (×6 H_2_O), 1 mM CaCl_2_ (×2 H_2_O), 15 mM glucose, 10 mM HEPES, pH 7.2). An inverted microscope (Olympus IX71) with a PlanApoN, 60, NA 1.42 oil immersion objective, 0.17/FN 26.5 (Olympus, KeyMed Ltd., Southend-on-Sea, UK) was used. The microscope was equipped with a CoolSNAP HQ2 monochrome camera (Photometrics) and a DV2 optical beam-splitter (MAG Biosystems, Photometrics). Images were acquired and processed using MetaFluor 7.1, (Meta Imaging Series, Molecular Devices). FRET changes were measured as a ratio of the acceptor fluorophore emission (545 nm) to donor emission (480 nm) (i.e., 545 nm/480 nm expressed as R/R_0_, where R is the intensity emission fluorescence ratio at time t, and R_0_ is the average emission fluorescence intensity ratio value of the last 8 frames taken before the addition of the stimulus).

### 2.5. Statistical Analysis

Statistical analysis was performed with GraphPad Prism 5.0. The number of technical and biological replicates is indicated in the figure legends. All groups that were statistically compared showed equal variance. Data is presented as mean ± s.e.m. One-way ANOVA with Bonferroni’post hoc correction or Student’s *t*-test were used as appropriate.

## 3. Results and Discussion

### 3.1. AKAP79 as Targeting Domain for the cAMP FRET-Based Sensor CUTie

AKAP79 binds PKA, which, when activated by cAMP, can phosphorylate and activate long isoforms of PDE4 [15,27] and can phosphorylate and inhibit AC5/6 [21,22] resulting in reduced levels of cAMP. Therefore, overexpression of an AKAP79-targeted sensor for cAMP can potentially affect the local level of cAMP detected and result in an underestimation of the cAMP signal that is generated in native conditions at the AKAP79 site.

**Figure 1:**
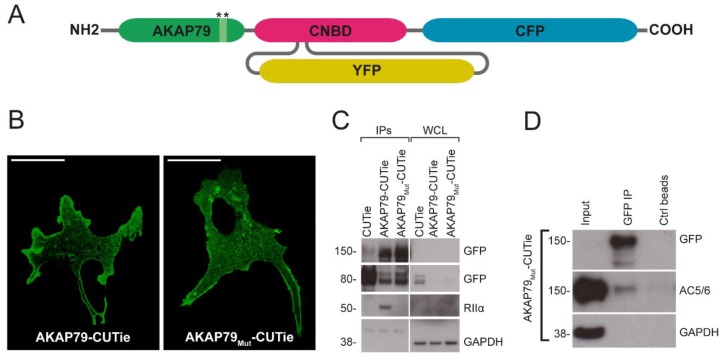

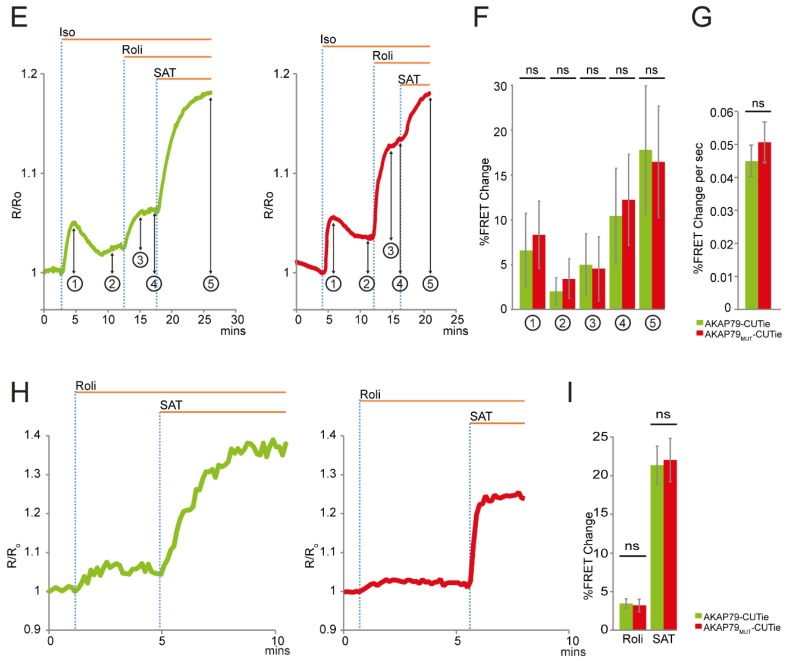
Effect on 3′–5′cyclic AMP (cAMP) readouts by using A-kinase anchoring protein 79 (AKAP79) as a targeting domain for CUTie. (**A**) Schematic representation of the targeted CUTie sensor. Stars represent the region where the mutations were introduced in AKAP79_Mut_-CUTie. CNBD is cyclic nucleotide binding domain, CFP is cyan fluorescent protein, and YFP is yellow fluorescent protein; (**B**) Confocal images showing the predominant localization of AKAP79-CUTie and AKAP79_Mut_-CUTie at the plasmalemma of neonatal rat ventricular myocytes (NRVMs). Scale bar is 10 μm; (**C**) Western blot analysis showing co-immunoprecipitation of protein kinase A (PKA) RIIα with AKAP79-CUTie and not with AKAP79_Mut_-CUTie. CUTie, the untargeted cytosolic version of the cAMP sensor [9], was used as the control. WCL indicates whole cell lysate; (**D**) Western blot analysis showing co-immunoprecipitation of AKAP79_Mut_-CUTie and AC5/6. Ctrl bead indicates pulldown with beads without the GFP-trap_A; Representative kinetics (**E**) and summary (**F**) of fluorescence resonance energy transfer (FRET) change on application of 0.5 nM Isoproterenol (Iso) followed by 10 μM Rolipram (Roli) in NRVMs expressing AKAP79-CUTie (green) and AKAP79_Mut_-CUTie (red), respectively. Bars in (**F**) were calculated as relative increase as indicated by the corresponding arrows in (**E**): ① is Iso maximal response; ② is Iso plateau; ③ is Roli after Iso plateau; ④ is Roli over basal; and ⑤ is saturating stimulus (SAT) of 25 μM forskolin + 100 μM IBMX; (**G**) Summary of the rate of FRET change on application of Iso 0.5 nM; Representative kinetics (**H**) and summary (**I**) of FRET change on application of Rolipram (Roli) 10 μM in absence of β-adrenergic receptors (β-AR) stimulation in NRVMs expressing AKAP79-CUTie (green) and AKAP79_Mut_-CUTie (red), respectively. SAT indicates application of saturating stimulus (25 μM forskolin + 100 μM IBMX). Statistical significance was assessed using one-way ANOVA with post hoc correction test except for G, where Student’s *t*-test was used. For all experimental sets, data is presented as mean ± s.e.m. For all experimental sets *n* ≥ 15 from at least three biological replicates (independent myocyte isolations).

To establish whether targeting the cAMP sensor via AKAP79 affects local levels of cAMP, we compared the FRET signal detected by the cAMP sensor AKAP79-CUTie [9] with the signal detected by a variant of this sensor (AKAP79_Mut_-CUTie) where the amphipathic helix that binds the D/D domain of the PKA RII subunit is disrupted by substituting two leucine for two proline residues (L391P and L392P) [17] (Figure 1A). When expressed in neonatal rat ventricular myocytes (NRVMs), both AKAP79-CUTie and AKAP79_Mut_-CUTie showed plasma membrane localization as expected (Figure 1B). Loss of RIIα-binding to AKAP79_Mut_-CUTie was confirmed by immunoprecipitation of the sensor and detection by western blot analysis of RII in the immunoprecipitate (Figure 1C). A band corresponding to the soluble sensor CUTie (~80 kDa) was detected in the lysates of cells expressing the CUTie-targeted probes (Figure 1C). This may be explained by the presence of a putative kozak sequence between the AKAP79 and the cyclic nucleotide binding domain (CNBD) moieties. Alternatively, it could be the consequence of protein degradation. As demonstrated previously for AKAP79-CUTie [9], integration of AKAP79_Mut_-CUTie within the expected macromolecular complex at the plasmalemma was confirmed by co-immunoprecipitation of the sensor and detection of endogenous AC5/6 in the immunoprecipitate (Figure 1D).

In order to determine whether the anchoring of PKA to AKAP79 may affect the amplitude and kinetics of the local cAMP signal, NRVMs expressing either AKAP79-CUTie or AKAP79_Mut_-CUTie were treated with the β-adrenergic agonist isoproterenol (Iso, 0.5 nM). A transient FRET change on application of the stimulus was detected with both sensors and no significant difference was apparent in the amplitude of the peak response or in the subsequent plateau level (Figure 1E,F). Similarly, there was no difference in the rate of the FRET change detected on application of Iso (Figure 1G). Similar results were found when FRET values were averaged over the entire cell or in the region of interest drawn in correspondence with the plasma membrane, excluding the possibility that the fraction of untargeted, soluble CUTie may affect the readings. These results indicate that the recruitment of PKA at the plasmalemma on overexpression of AKAP79-CUTie does not affect the local cAMP response to β-adrenergic receptor stimulation.

In order to reveal any effect of local PKA recruitment on the extent of PDE4 activity, the specific PDE4 inhibitor rolipram (10 μM) was applied in the presence of Iso. As shown in Figure 1E,F, the wild-type and mutant sensors detected a similar increase in local cAMP level on inhibition of PDE4. AKAP79-CUTie and AKAP79_Mut_-CUTie generated a comparable FRET change also at saturating cAMP, as obtained by application of 25 μM forskolin and 100 μM IBMX (SAT), confirming that the two sensors respond with similar FRET change to maximal activation.

For the experiments described above, the NRVMs are kept in culture for two days to allow expression of the sensor. It is therefore possible that overexpression of the AKAP79-targeted reporter and its associated PKA may result in phosphorylation of PDE4 during this time in culture. To assess whether overexpression of AKAP79-CUTie had an effect on the activity of PDE4 at baseline, we measured the amplitude of the cAMP response detected by AKAP79-CUTie or AKAP79_Mut_-CUTie on PDE4 inhibition but in the absence of β-adrenergic stimulus. As shown in Figure 1H,I, application of rolipram (10 μM) resulted in a similar FRET change for the two sensors. These results indicate that the anchoring of PKA to the targeting domain AKAP79 has no impact on local cAMP levels.

### 3.2. AKAP79 as a Targeting Domain for the PKA Activity FRET-Based Sensor AKAR4

We next sought to establish whether the anchoring of PKA to the AKAP79 targeting domain may affect the level of locally detected PKA mediated phosphorylation.

**Figure 2:**
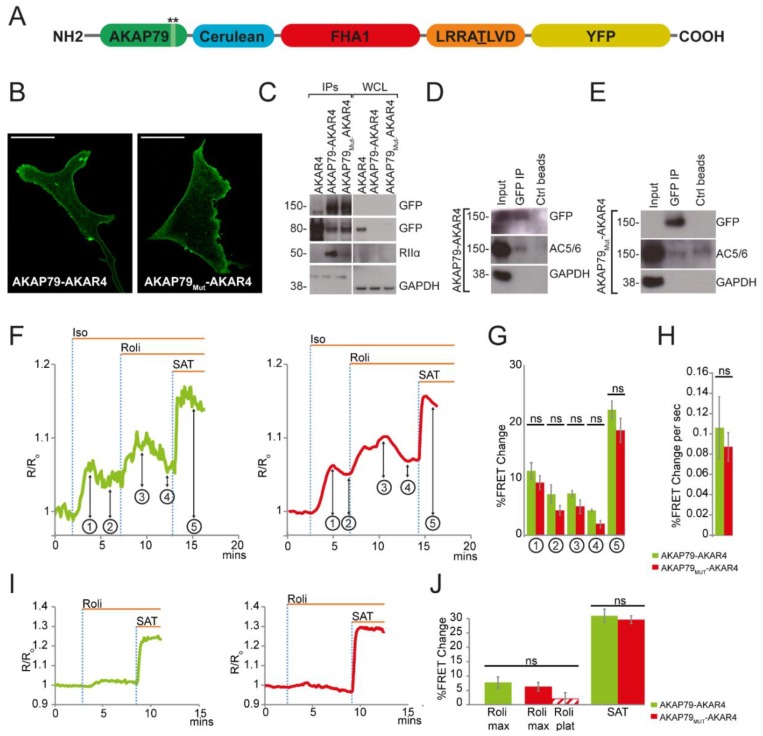

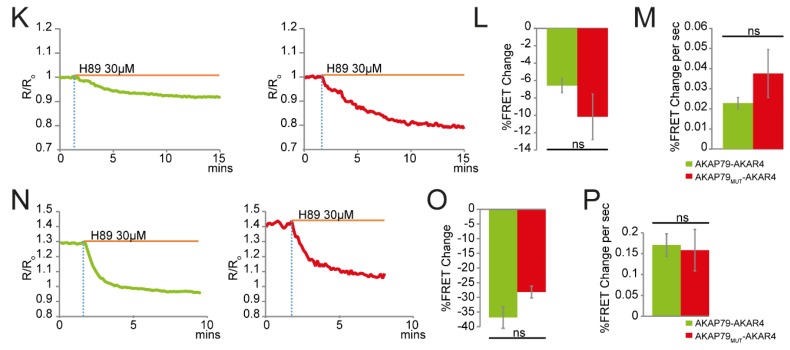
Effect of using AKAP79 as a targeting domain for AKAR4 on detection of PKA activity. (**A**) Schematic representation of the targeted AKAR4 sensor. Stars indicate the region where the mutations were introduced in AKAP79_Mut_-AKAR4. FHA1 is the phospho-amino acid binding domain. LRRATLVD is the PKA phosphorylation consensus sequence domain. Cerulean is the cyan fluorescent protein and YFP is the yellow fluorescent protein; (**B**) Confocal images showing the predominant localization of AKAP79-AKAR4 and AKAP79_Mut_-AKAR4 at the plasmalemma of NRVMs. Scale bar is 10 μm; (**C**) Western blot analysis showing co-immunoprecipitation of PKA RIIα with AKAP79-AKAR4 and AKAP79_Mut_-AKAR4 in NRVMs. WCL is whole cell lysate. AKAR4 [29], a cytosolic version of the PKA activity sensor, was used as a control; Western blot analysis showing co-immunoprecipitation of AKAP79-AKAR4 (**D**) and AKAP79_Mut_-AKAR4 (**E**) with endogenous AC5/6 in NRVMs. Ctrl beads indicate the pulldown with beads without the GFP-trap_A; Representative kinetics (**F**) and summary of amplitude (**G**) of FRET change on application of 0.05 nM Isoproterenol (Iso) followed by Rolipram (Roli) 10 μM in NRVMs expressing either AKAP79-AKAR4 (green) or AKAP79_Mut_-AKAR4 (red). ① is Iso max; ② is Iso plateau; ③ is Roli max after iso plateau; ④ is Roli plateau over Iso plateau; and ⑤ is saturating stimulus (SAT) of 25 μM forskolin + 100 μM IBMX; (**H**) Summary of the rate of FRET change on application of isoproterenol. For experiments shown in (**F**–**H**), *n* ≥ 13; Representative kinetics (**I**) and summary (**J**) of FRET change on application of Roli (10 μM) in NRVMs expressing either AKAP79-AKAR4 or AKAP79_Mut_-AKAR4. Striped bar in red represents plateau level of PKA activity reached on decay of the signal after the peak response. SAT indicates saturating stimulus (forskolin 25 μM + IBMX 100 μM). *n* ≥ 18; Representative kinetics (**K**) and summary of amplitude (**L**) and rate (**M**) of FRET change on application of H89 30 μM to otherwise unstimulated cells. *n* ≥ 12; Representative kinetics (**N**) and summary of amplitude (**O**) and kinetics (**P**) of FRET change on application of H89 30 μM after maximal PKA activation in NRVMs expressing AKAP79-AKAR4 (in green) and AKAP79_Mut_-AKAR4 (in red). *n* ≥ 8. For all datasets, at least three biological replicates (independent myocyte isolations). For all experimental sets, data is presented as mean ± s.e.m. For statistical analysis, Student’s *t*-test was used except for G, where one-way ANOVA with post hoc correction test was used.

For these experiments wild-type AKAP79 or the mutant AKAP79 were fused to the PKA activity reporter AKAR4 [29] (Figure 2A). Expression of AKAP79-AKAR4 and AKAP79_Mut_-AKAR4 in NRVMs showed the expected localization at the plasmalemma (Figure 2B). Disruption of PKA RIIα-binding to AKAP79_Mut_-AKAR4 was confirmed by immunoprecipitation of the sensor and detection by western blot analysis of RII in the immunoprecipitate. The results show that while AKAP79-AKAR4 co-immunoprecipitates PKA RII, only traces of RII can be detected in the AKAP79_Mut_-AKAR4 immunoprecipitate (Figure 2C). Because AKAP79 can dimerize [31], the residual presence of RII subunits in the AKAP79_Mut_-AKAR4 may be due to interaction of the sensor with endogenous wild-type AKAP79. The absence of PKA RII in whole cell lysate (Figure 2C) can be explained by the fact that only a small fraction of the whole cell lysate was loaded for these samples.

As for the targeted CUTie reporters, untargeted cytosolic AKAR4 (~80 kDa) can be detected in the lysate of cells expressing AKAP79-targeted AKAR4 sensors (Figure 2C), which may be due to the presence of a putative kozak sequence between the AKAP79 and the Cerulean moieties. Again, no difference was found when comparing FRET values calculated over the entire cell or exclusively at the plasma membrane, confirming also in this case that the fraction of cytosolic AKAR4 does not impact the readings. Integration of AKAP79-AKAR4 and AKAP79_Mut_-AKAR4 within the expected macromolecular complex was confirmed by co-immunoprecipitation of the respective sensor with endogenous AC5/6 (Figure 2D,E).

To assess the effect of β-AR stimulation on local PKA activity, cells were challenged with 0.05 nM Iso. This reduced concentration of agonist was necessary to avoid sensor saturation on subsequent application of rolipram and saturating stimulus. NRVMs expressing either AKAP79-AKAR4 or AKAP79_Mut_-AKAR4 showed a similar amplitude (Figure 2F,G) and rate (Figure 2F,H) of FRET change on agonist application. In both cases, the majority of cells showed a transient response to Iso (90.91% and 84.62% for AKAP79-AKAR4 and AKAP79_Mut_-AKAR4, respectively). The plateau value reached after the peak response did not show significant difference between the two sensors. In addition, no difference between the increase in PKA-mediated phosphorylation was detected when PDE4 was selectively inhibited with rolipram (10 μM) after β-AR stimulation (Figure 2F,G).

To assess any effect of overexpression of the targeted AKAR4 reporters at baseline, 10 μM rolipram was applied in the absence of other stimuli. As shown in Figure 2I,J, no significant difference in the level of PKA activity was apparent; however, 47% of NRVMs expressing AKAP79_Mut_-AKAR4 showed a transient response to the inhibitor, while this was not observed in any of the cells expressing AKAP79-AKAR4.

The transient response on application of rolipram may be explained by higher phosphatase activity at baseline in the cells expressing the AKAP79_Mut_-AKAR4 sensor. To test this hypothesis, NRVMs expressing AKAP79-AKAR4 or AKAP79_Mut_-AKAR4 were either treated with the PKA inhibitor H89 (30 μM) at baseline (Figure 2K–M) or with a saturating stimulus before the addition of H89 (30 μM) (Figure 2N–P). In the absence of kinase activity, the FRET signal is expected to decrease as the sensor is dephosphorylated by phosphatases. As shown in Figure 2K–P, there was no statistically significant difference in the extent of dephosphorylation of AKAP79-AKAR4 and AKAP79_Mut_-AKAR4, both in terms of amplitude and in terms of rate of dephosphorylation. These findings indicate that both at baseline and on maximal activation of PKA, respectively, the local phosphatase activity is not affected by overexpression of AKAP79-AKAR4. A possible explanation for the transient response to PDE4 inhibition detected by AKAP79_Mut_-AKAR4 is that in the presence of a small increase in cAMP, as elicited by application of rolipram, local phosphatases can more easily counteract the phosphorylation brought about by the reduced amount of PKA recruited at AKAP79 complexes in cells expressing AKAP79_Mut_-AKAR4 than in cells expressing AKAP79-AKAR4.

In conclusion, our studies demonstrate that the ability of AKAP79-targeted sensors to locally recruit PKA to AKAP79-specific plasmalemma nanodomains does not significantly affect local cAMP/PKA signaling. Therefore, these targeted sensors are expected to accurately report local signals as they happen in wild-type cells.
